# The function of the two-pore channel TPC1 depends on dimerization of its carboxy-terminal helix

**DOI:** 10.1007/s00018-016-2131-3

**Published:** 2016-01-18

**Authors:** Nina Larisch, Sonja A. Kirsch, Alexandra Schambony, Tanja Studtrucker, Rainer A. Böckmann, Petra Dietrich

**Affiliations:** 1 Molecular Plant Physiology, Department of Biology, University of Erlangen-Nürnberg, Staudtstrasse 5, 91058 Erlangen, Germany; 2 Computational Biology, Department of Biology, University of Erlangen-Nürnberg, Staudtstrasse 5, 91058 Erlangen, Germany; 3 Developmental Biology, Department of Biology, University of Erlangen-Nürnberg, Staudtstrasse 5, 91058 Erlangen, Germany

**Keywords:** Vacuole, Calcium signaling, MD simulation, Patch-clamp, Microscale thermophoresis, Mutation

## Abstract

Two-pore channels (TPCs) constitute a family of intracellular cation channels with diverse permeation properties and functions in animals and plants. In the model plant *Arabidopsis*, the vacuolar cation channel TPC1 is involved in propagation of calcium waves and in cation homeostasis. Here, we discovered that the dimerization of a predicted helix within the carboxyl-terminus (CTH) is essential for the activity of TPC1. Bimolecular fluorescence complementation and co-immunoprecipitation demonstrated the interaction of the two *C*-termini and pointed towards the involvement of the CTH in this process. Synthetic CTH peptides dimerized with a dissociation constant of 3.9 µM. Disruption of this domain in TPC1 either by deletion or point mutations impeded the dimerization and cation transport. The homo-dimerization of the CTH was analyzed in silico using coarse-grained molecular dynamics (MD) simulations for the study of aggregation, followed by atomistic MD simulations. The simulations revealed that the helical region of the wild type, but not a mutated CTH forms a highly stable, antiparallel dimer with characteristics of a coiled-coil. We propose that the voltage- and Ca^2+^-sensitive conformation of TPC1 depends on *C*-terminal dimerization, adding an additional layer to the complex regulation of two-pore cation channels.

**Electronic supplementary material:**

The online version of this article (doi:10.1007/s00018-016-2131-3) contains supplementary material, which is available to authorized users.

## Introduction

Two-pore cation channels (TPCs) are intracellular ion channels residing in the vacuolar membrane of plant cells, and in lysosomes and endosomes of mammalian cells [1, 2]. Depending on their biophysical properties and host species, they play diverse roles, ranging from cation and pH homeostasis, sensing of the metabolic state, cell-to-cell signaling, control of the membrane potential and membrane trafficking to pigmentation, and even to the control of Ebola virus invasion in animal cells [1–8]. The underlying molecular mechanisms are in most cases fairly unknown.

TPCs belong to the superfamily of voltage-gated ion channels that are built from Shaker-like domains of 6 transmembrane segments (S1-S6) and a pore-forming helix between S5 and S6. One TPC monomer contains two Shaker-like domains connected by a cytosolic linker, and the channel functions as a dimer [1, 9, 10]. The cytosolic *N*-terminus contains a dileucine motif, which is responsible for targeting of the channel to the tonoplast in plant cells or lysosomes in animal cells [11, 12]. Two EF-hand motifs in the linker domain of plant, but not animal TPCs mediate binding and channel activation by cytosolic calcium ions [13, 14]. In contrast, animal TPC1 and TPC2 are directly activated by phosphatidyl-inositol-3,5-bisphosphate PI(3,5)P_2_ [6, 7, 15] and have been identified as mediators of nicotinic acid adenine dinucleotide phosphate (NAADP)-induced Ca^2+^-release from endolysosomes [9, 12, 16–18].

While animal cells express two or more different channels, in most plant species there is only one TPC isoform, TPC1 [1, 2]. TPC1 encodes the slow vacuolar (SV) channel [19] and mediates the passage of K^+^ and Na^+^, and other monovalent as well as divalent cations, although a direct involvement in calcium release from the vacuole in vivo is strongly debated [20–25].

Recently, a role for TPC1 in a rapid, long-distance signaling system based on Ca^2+^ waves has been uncovered [3]. In response to a localized salt stimulus at the *Arabidopsis* root, the calcium wave mainly travels through the cortex and endodermis at speeds of up to 420 μm/s, and is required for activation of stress responsive genes in the shoot. Lack of TPC1 largely reduces the travel speed of this trigger wave, while *TPC1* overexpression accelerates it. TPC1 thus plays an important role in long-distance signaling in response to salt stress [3]. How the density or distribution of TPC1 in the vacuolar membrane affects the speed of the long-distance calcium wave remains largely unknown.

Deregulation of the voltage-dependent activity of TPC1 leads to imbalanced cation homeostasis in the vacuole of *fou2*, which has a threefold higher Ca/K ratio as compared to the wild type [26]. The expression profile of *fou2* resembles that of wild type plants under K starvation, and *fou2* plants produce more oxylipins in response to wounding [27, 28]. The *fou2/*TPC1D454 N mutation introduces an amino acid exchange in the binding site for luminal Ca^2+^, which abolishes the inhibition of TPC1 by luminal calcium ions and shifts the activity range towards more negative potentials [26, 29]. These results demonstrate the important role of TPC1 for cation homeostasis and vacuolar storage function.

A tight regulation of SV channels prevents loss of potassium and other cations from the vacuole, and many factors down-regulating or blocking TPC1 have been identified, including luminal calcium ions [30], protons [31], sodium ions [32], and polyamines [33]. A further negative regulation is mediated by polyunsaturated fatty acids, which like luminal Ca^2+^ ions shift the voltage dependence towards more negative potentials [34]. In comparison to these ionic and metabolic factors, less is known about interactions of TPC1 with regulatory proteins and their sites of interaction. 14-3-3 proteins rapidly reduce SV currents [35, 36], and regulation by kinases and phosphatases is most likely [37, 38].

Except for a few cases like calcium binding [13, 29] or block by polyamines [33], little is known about the structure function relation in TPC1. Here, we report that the predicted carboxy-terminal helical regions of two TPC1 monomers are prone to dimerization. These dimers are shown to be essential for the function of the (dimerized) TPC1 channel. We employed wild type coarse-grained and atomistic simulations, as well as coarse-grained mutant simulations, which revealed that the wild type *C*-terminal domain forms a stable antiparallel coiled-coil. In contrast, the mutant showed a highly promiscuous dimerization pattern, pointing to a significantly decreased affinity. We suggest that the dimerization of the wild type TPC1 carboxyl-termini stabilizes the channel in a conformation, which is sensitive to Ca^2+^-binding and depolarization, adding an additional layer to the complex regulation of two-pore cation channels.

## Materials and methods

### Plant material and growth conditions

*Arabidopsis thaliana* Col-0 wild type and *tpc1*-*2* mutant plants [20] were used. After seed stratification at 4 °C for 3 days, plants were grown on soil in a growth chamber under 8 h light/16 h dark conditions at 22 °C. *Nicotiana benthamiana* wild type plants were cultivated on soil in the greenhouse at 22 °C under a 16 h light/8 h dark cycle and used after 6 weeks for infiltration.

### Cloning procedures

Cloning procedures for *C*-terminal GFP-fusions are described elsewhere [11], and eGFP was used in all cases. PCR-primers were designed accordingly with the respective cloning sites and with or without stop-codon and purchased from Sigma. For construct details and primer sequences see Supplemental Table 1 and 2.

For BiFC analyses the gateway entry vector pENTR-D-TOPO and the destination vectors pDEST–^GW^VYCE and pDEST–^GW^VYNE were used for *C*-terminal fusion of the Venus *C*- and *N*-terminus, respectively, and pDEST–VYCE(R)^GW^ and pDEST–VYNE(R)^GW^ for *N*-terminal tagging [39].

For the Co-IP tests the *C*-terminally tagged GFP-fusions were used as templates to amplify the soluble *C*-terminus (amino acids 673–733) of TPC1 or TPC1-3LP fused to GFP with PCR. The constructs were subcloned into the pENTR-D-TOPO vector and afterwards brought into the expression vectors with EcoRI and XhoI (New England Biolabs), so that they were *N*-terminally tagged either with 6x Myc (pCS2+MT) or with the FLAG-tag (pCS2 + Flag) [40].

### Protoplast isolation and transformation

Expression of GFP-fusion proteins for electrophysiological measurements or confocal microscopy was performed in *A. thaliana tpc1*-*2* mesophyll protoplasts as described [11, 13]. Briefly, leaves were enzymatically digested to release mesophyll protoplasts, which were then transformed by the polyethylene glycol method. Protoplasts were used for electrophysiological measurements and subcellular localization of GFP fluorescence 2–4 days after transformation.

### Confocal microscopy

Fluorescence signals were detected and documented with a TCS SP2 confocal laser scanning microscope and the Leica Confocal Software (Leica Microsystems). A 488 nm Argon laser was used for excitation of GFP and of the auto-fluorescence of chlorophyll. GFP-signals were detected from 500 to 556 nm and chlorophyll signals from 675 to 767 nm. Venus was excited with a 543 nm helium–neon laser and detected in the range from 520 to 556 nm, the corresponding chlorophyll signals were detected from 637 to 736 nm. Images were processed with Photoshop (Adobe Systems).

### Electrophysiological recordings

Patch-clamp experiments were performed on transformed vacuoles harboring two-pore channels as identified by GFP fluorescence. The pipette (luminal) solution consisted of 100 mM K-gluconate, 2 mM MgCl_2_, 10 mM EGTA, 10 mM MES, pH 5.5/Tris. The bath (cytosolic) solution contained 50 mM K-gluconate, 1 mM CaCl_2_, 1 mM MgCl_2_, 2 mM DTT, 10 mM HEPES, pH 7.5/Tris. Solutions were adjusted to 430 mosmol/kg by adding D-sorbitol. For analysis of the TPC1-S706 mutants two further calcium concentrations of the bath solution were used in which 1 mM CaCl_2_ was substituted by 0.2 mM or 0.05 mM CaCl_2_, respectively. Competition assays using the CTH peptide were performed on endogenous TPC1 channels in vacuoles isolated from Col-0 plants. Peptides were dissolved at 10 mM in bath solution supplemented with 1 % DMSO and 0.1 % pluronic, and diluted >600-fold for application in patch-clamp recordings. Currents were recorded either in the whole-vacuolar or cytosolic side-out configuration of the patch-clamp technique, using an EPC10 amplifier and the program PULSE (HEKA electronics, Lambrecht, Germany). Recordings and data analysis were performed as described earlier [11]. Relative open probabilities (*P*_o_) were determined from tail currents at −53 mV, following pulses to the different test voltages. Tail currents were normalized to the maximum values at 1 mM Ca^2+^, plotted as a function of the applied test voltage, and fitted according to the Boltzmann equation:

$$P_{O} = \frac{A}{{1 + exp^{{\frac{zF}{RT} \cdot (V_{o} - V)}} }}$$

where *V*_o_ and *z* are parameters for the voltage at half-maximum open probability and the apparent number of gating charges, respectively, and *A* describes the maximum conductance that is reached under the experimental conditions. For comparison of the voltage dependence in the presence of different calcium concentrations, the gating charge as determined for TPC1 in the presence of 1 mM Ca^2+^ was held constant for fits at lower Ca^2+^, as this parameter does not dependent on the cytosolic Ca^2+^ concentration [23].

### BiFC, tobacco infiltration, and fluorescence quantification

BiFC studies were performed as described [41] using a Split Venus system [39]. The Agrobacterium strain C58C1 [42] harboring the plasmid of interest and the helper strain p19 [43] were grown in LB medium supplemented with 100 µg/ml Kanamycin at 29 °C overnight. The cultures were brought to an optical density (OD_600_) of 1.0 in infiltration buffer (10 mM MES, 10 mM MgCl_2_, 100 µM acetosyringone, pH 5.7/KOH) and the combinations to test for co-expression mixed at equal amounts. The p19 helper strain (OD_600_ = 1.0) was added to these mixtures in a 1:1 ratio. Suspensions were incubated for 2 h before infiltration into the abaxial side of 6-week-old *N. benthamiana* leaves. Each leaf was infiltrated with several plasmid combinations and a positive control (TPC1/pDEST-^GW^VYCE and TPC1/pDEST-^GW^VYNE) as well as a negative control (p19 alone) at separated areas to monitor comparability of the transient expression and to define background fluorescence.

For fluorescence quantification leaf disks (Ø 6 mm) were cut out of the infiltrated areas with a cork borer 2.5 day after infiltration. Leaf disks were placed upside-down in a black 96-well plate prefilled with 70 µl H_2_O per well to minimize dehydration effects. Fluorescence was measured with a plate-reader (Infinite F200, Tecan) with an excitation wavelength of 485 ± 20 nm and an emission wavelength of 525 ± 25 nm [41].

### Cell culture, transfection, Co-IP and western blot analysis

HEK293T cells were propagated in DMEM GIBCO Glutamax (Life Technologies) supplemented with 10 % fetal calf serum. Cells were seeded in 6–well plates (20–30 % confluency) and transfected the next day using 10 µl Roti-Fect (Carl Roth) per 2 µg total DNA (1 µg DNA per plasmid). Two days after transfection cells were washed with 2 ml PBS buffer (Life Technologies) and harvested for immunoprecipitation in 150 µl cold lysis buffer (10 mM HEPES, 150 mM NaCl, 1 % Nonidet P40, 5 % glycerol, pH 7,4/KOH) supplemented with protease inhibitors and phosphatase inhibitors (Complete mini EDTA free and PhosStop, Roche). Cells were disrupted mechanically with a syringe (cannula diameter 0.55 mm). The lysate was cleared by centrifugation at 13.500 rpm and 4 °C for 10 min. Total protein concentrations were measured with a bicinchoninic acid protein assay (Applichem), typically the total protein amount was 500–600 µg. Loading controls of 50 µg of the lysate were taken accordingly and filled up to 20 µl with lysis buffer before adding 5 µl of 5x sample buffer (250 mM Tris, 5 % SDS, 25 % glycerol, 0.25 % bromophenol blue, 250 mM β–mercaptoethanol, pH 6.8/HCl) and boiling. For immunoprecipitation, 3 µg of mouse anti-c-Myc Epitope antibody 9E10 (sc-40, Santa Cruz) was used per sample. Samples were incubated with the antibody for 45 min at 4 °C with a rotator, then 10 µl of protein G Dynabeads (Life Technologies) equilibrated in the lysis buffer was added to each sample and incubation continued for another 45 min. Samples were washed three times with cold lysis buffer before eluting three times with 20 µl of 100 mM glycine (pH 2.5/HCl). After neutralizing the pH 15 µl of 5x sample buffer was added and samples were boiled.

Proteins of the loading controls and samples were separated with SDS-PAGE (12 % gel) and transferred to a PVDF membrane in duplicate. Membranes were blocked for 1 h with blocking reagent (Roche) and incubated over night at 4 °C with rabbit anti-Myc or anti-Flag antibodies. After washing three times with TBST buffer, the membranes were incubated for 1 h at RT with anti-rabbit antibody coupled to alkaline phosphatase. Membranes were washed three times with TBST, followed by two times with alkaline phosphatase buffer (100 mM NaCl, 100 mM Tris, pH 9.5/HCl). Colorimetric detection of the proteins was performed with NBT/BCIP substrate (Roche) in alkaline phosphatase buffer until a sufficient staining was achieved. All antibodies used for the western blots were purchased from Cell Signaling Technology.

### Molecular dynamics simulations

All simulations were carried out using GROMACS 4.6.x [44]. The secondary structure of the TPC1 *C*-terminal part (679–733) was addressed applying different prediction tools (Table 1). The consensus helical sequence RSQRVDTLLHHMLGDEL, i.e., those residues that were predicted by at least three out of four prediction tools, was converted into an α-helical structure using PyMOL [45], and the atomistic structure was changed to coarse-grained representation with the aid of *martinize* [46]. Aggregation of two TPC helices was studied from 100 simulations with randomized starting structures, using the previously developed DAFT algorithm [47], in conjunction with the polarizable Martini [48] force field version 2.2; the two α-helices were put in a dodecahedron simulation box at a specified distance to each other. Finally, the solvent was added using *insane* [49, 50]. In this manner, each generated system contained two 17 amino acids long helices and approximately 5300 polarizable water beads. The usage of a polarizable water model instead of a standard water model was shown to be more appropriate for adsorption studies of small peptides to a bilayer since the standard water resulted in overestimation of binding, probably due to a difference in electrostatic screening [51]. In addition to the wild type TPC1 systems, coarse-grained systems of the TPC1-3LA mutant were set up in the same manner as described above and simulations were performed under the same simulation conditions.

**Table 1:**
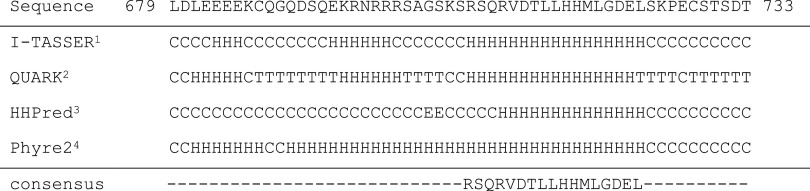
Secondary structure prediction of the TPC1 carboxyl-terminus

*C* Coil, *H* helical, *E* sheet, *T* beta-turn

^a^Yang et al. [85], Roy et al. [86], Zhang [87]

^b^Xu, Zhang [88]

^c^Remmert et al. [89], Söding [90], Söding et al. [91]

^d^Kelley, Sternberg [92]

Five atomistic [force field Amber 14SB, 52] starting configurations were generated from selected coarse-grained frames of the wild type simulations using the *backward* method [53]. The K^+^-ion concentration of 100 mM was chosen similar to the cytoplasmic salt concentrations in plants [54]. Additionally, the unit cells were altered to smaller ones, containing approximately 5700 TIP3P [55] water molecules as well as counterions (Cl^−^).

The modeled sequence is a small part of a larger protein sequence. To describe the helices accurately, the termini were kept neutral in all coarse-grained simulations or acetylated and amidated, respectively, in the atomistic simulations.

### Simulation details

After automatically generating each 100 coarse-grained (CG) starting structures using DAFT, the wild type and mutant systems were subjected to a relaxation and equilibration process using *martinate* [56]. The energy was minimized using steepest-descent (500 steps) and a 10 ps position-restrained simulation with a time step of 2 fs. Subsequently, the systems were relaxed in a 100 ps *NpT* simulation with an integration time step of 20 fs under constant pressure *p* and temperature *T*. In all simulations, the isotropic pressure of 1 bar was kept constant using a weak coupling scheme [57] with a 3 ps time constant. The temperature was maintained at 310 K with the v-rescale thermostat and a time constant of 1 ps [58]. The relative dielectric constant was globally set to 2.5 and long-range electrostatic interactions were treated using particle-mesh Ewald [59] summation with a real-space cutoff of 1.2 nm. Dispersion interactions were described by a Lennard-Jones 12–6 potential that was shifted to zero between 0.9 nm and 1.2 nm. The production simulations were run for 250 ns each, using a time step of 20 fs.

Selected backmapped coarse-grained (CG) structures of the wild type were studied at atomistic resolution at 300 K and 1 bar using the Amber14SB force field. The simulation length was 200 ns (4 simulations) and 270 ns (1 simulation), respectively, with an integration time step of 2 fs. The temperature was kept constant using the Nosé-Hoover [60, 61] thermostat with a time constant of 0.5 ps. The pressure was modulated isotropically with the Parinello-Rahman [62] barostat and a time constant of 10 ps. The relative permittivity was set to 1. Long-range Coulomb interactions were calculated with the particle-mesh Ewald summation and a real-space cutoff of 1 nm. Van der Waals interactions were treated with a cutoff of 1 nm as typical for Amber force fields [63]. Furthermore, long-range dispersion corrections were applied for the energy and pressure.

### Data analysis

Relative orientations of the *C*-terminal helices of AtTPC1 and mutated AtTPC1-3LA were described using Euler angles (Fig. 7b), which characterize the relative orientation of two peptides [47, 64]. The matrix with Euler angles was obtained by least-square fitting a structure on a reference structure. To investigate the stability of formed dimers, the tilt angle between the dimers was determined over the last 50 ns of each simulation and its distribution plotted in a histogram. The tilt, ranging from 0° to 180°, was then divided into three subintervals (0°–50°, 50°–130°, and 130°–180°) in the case of wild type dimers and four subintervals (0°–35°, 35°–100°, 100°–150°, and 150°–180°) in the case of mutant dimers. These intervals were chosen according to the minima in the histogram (Fig. S6a). Subsequently, the number of transitions between these intervals was counted for each simulation and further evaluated.

### MicroScale thermophoresis (MST) binding assay

For MST experiments, peptides corresponding to the consensus helical sequence (RSQRVDTLLHHMLGDEL) and the 3LA mutant (RSQRVDTAAHHMAGDEL) were synthesized (Peptide Speciality Laboratories GmbH, Heidelberg, Germany). To allow for label-free MST binding experiments, additional wild type and mutant peptides were synthesized, which were *C*-terminally extended by addition of two tryptophanes and a short linker (AAWW).

Labelfree MicroScale Thermophoresis binding experiments were performed in cooperation with the 2bind GmbH (Regensburg, Germany), using 750 nM tryptophane containing target peptide in PBS pH 7.5, 1 % DMSO, 0.1 % Pluronic with varied concentrations of the ligand peptide at 80 % MST power, 100 % LED power in hydrophilic zero background capillaries on a Monolith NT.labelfree device at 25 °C (NanoTemper Technologies, Munich, Germany). Normalized fluorescence data sets (WT peptide and MT peptide) were analyzed in the thermophoresis and temperature jump. For determination of the binding affinity (*K*_D_) of the wild type peptide, the recorded fluorescence was normalized to the fraction bound (0 = unbound, 1 = bound), and fitted using the *K*_D_ fit formula derived from the law of mass action. Technical duplicates were performed for each experimental setup.

### Sequence information

Sequence data from this article can be found in the EMBL/GenBank data libraries under the following accession numbers: *Aly*-*Arabidopsis lyrata* (D7M2M4); *Ath*-*Arabidopsis thaliana* (B9DFD5); *Bna*-*Brassica napus* (A0A078G686); *Bol*-*Brassica oleracea* (A0A0D3E0B2); *Cru*-*Capsella rubella* (R0GT49); *Csa*-*Cucumis sativus* (A0A0A0K5Q7); *Egr*-*Eucalyptus grandis* (A0A059A094); *Gma*-*Glycine max* (1M3S8); *Gso*-*Glycine soja*(A0A0B2R1M3); *Hvu*-*Hordeum vulgare* (Q6S5H8); *Jcu*-*Jatropha curcas* (A0A067JJP4); *Mtr*-*Medicago truncatula* (A0A072VBZ7); *Nta*-*Nicotiana tabacum* (Q75VR1); *Osa*-*Oryza sativa* (Q5QM84); *Ptr*-*Populus trichocarpa* (U5FYB3); *Sit*-*Setaria italica* (K3XEV7); *Sly*-*Solanum lycopersicum* (K4CFU2); *Tae*-*Triticum aestivum* (Q6YLX9); *Tca*-*Theobroma cacao* (A0A061E309); *Zma*-*Zea mays* (B6SP34).

## Results

### A *C*-terminal region is essential for TPC1 function

We previously reported that deletion of the last 55 amino acids of AtTPC1, corresponding to the cytosolic carboxyl-terminus, resulted in a mutant (TPCΔC) which is correctly targeted to the tonoplast, but lacks channel function [11]. This indicates an essential role of this region for the activity of TPC1, but not for the targeting or trafficking process.

**Fig. 1:**
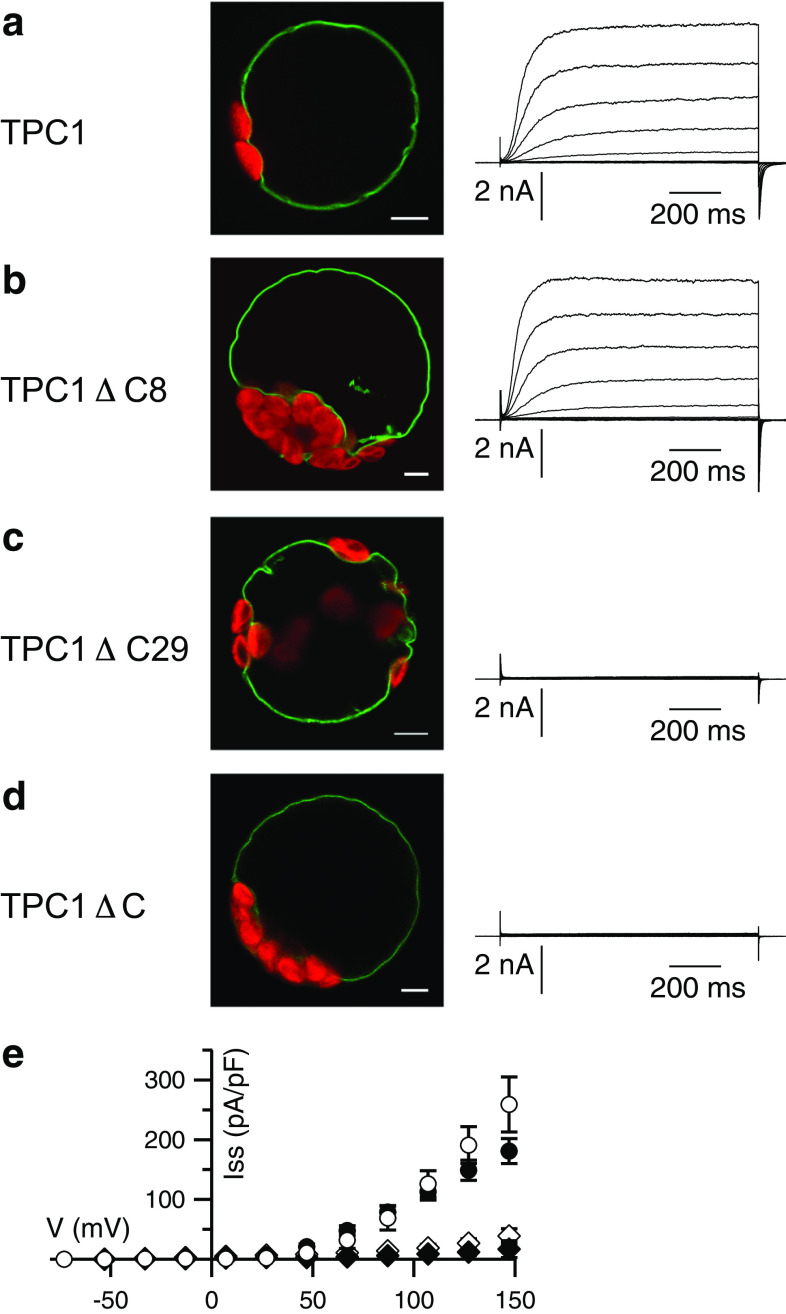
The *C*-terminus is essential for the function of TPC1. **a**–**d** Confocal fluorescence overlay images of the GFP (*green*) and chlorophyll (*red*) signals (scales represent 5 μm), and representative whole-vacuolar current responses of TPC1-GFP variants as indicated on the left. Applied voltages ranged from −73 to +147 mV in 20 mV intervals, starting from a holding potential of −53 mV. **e** Current–voltage relations of whole-vacuolar steady-state currents determined from traces as shown in (**a**–**d**) for TPC1-GFP (*closed circles*, *n* = 4), TPC1ΔC8-GFP (*open circles*, *n* = 5), TPC1ΔC29-GFP (*open rhombi*, *n* = 6), and TPC1ΔC-GFP (*closed rhombi*, *n* = 4) normalized to the vacuolar membrane capacitance. Data represent mean values ± SE

These previous results were obtained from electrophysiological recordings of excised patches and thus, due to the small membrane area, a little residual activity of TPC1ΔC may have escaped observation. We now compared slow vacuolar (SV) currents in the whole-vacuolar configuration of the patch-clamp technique after expression of wild type TPC1 and the TPC1ΔC mutant, respectively. Both channel variants were expressed as GFP-fusions in the *tpc1*-*2* knockout background, allowing localization and electrophysiological analysis of the introduced TPC1 versions as homo-dimers [11, 13]. In the presence of 1 mM CaCl_2_ in the bath solution, which fully activates the TPC1 wild type (Fig. 1a), no currents were obtained from vacuoles expressing the truncated channel (Fig. 1d). This result supports the conclusion that the *C*-terminus is necessary for the voltage- and Ca^2+^-dependent activity of TPC1 [11].

To investigate whether a specific sub-region of the carboxyl-terminus is involved in this regulation, two additional truncated channel versions were created: TPC1ΔC8 contained the residues 1–725, and TPC1ΔC29 the residues 1–704. Both mutants were localized in the tonoplast, indicating their expression, efficient ER export, and correct targeting (Fig. 1b, c). The lack of the last 8 amino acids did not interfere with the activity of TPC1ΔC8 (Fig. 1b), and the amplitudes and current–voltage behavior of the mutant were wild type like (Fig. 1b, e). In contrast, although TPC1ΔC29 was expressed normally (Fig. 1c, Fig. S1), this mutant stayed silent like the TPC1ΔC mutant lacking the whole carboxyl-terminus (Fig. 1c–e). These results identified amino acids 705–725 to include a region indispensable for channel function.

### Mutation in a predicted helical domain abolishes TPC1 activity

So far, no structural information is available about TPCs of plant or animal origin. To address the secondary structure of the *C*-terminus, different prediction tools were applied, which revealed a consensus helical sequence to span amino acids 707–723 (Table 1).

**Fig. 2:**
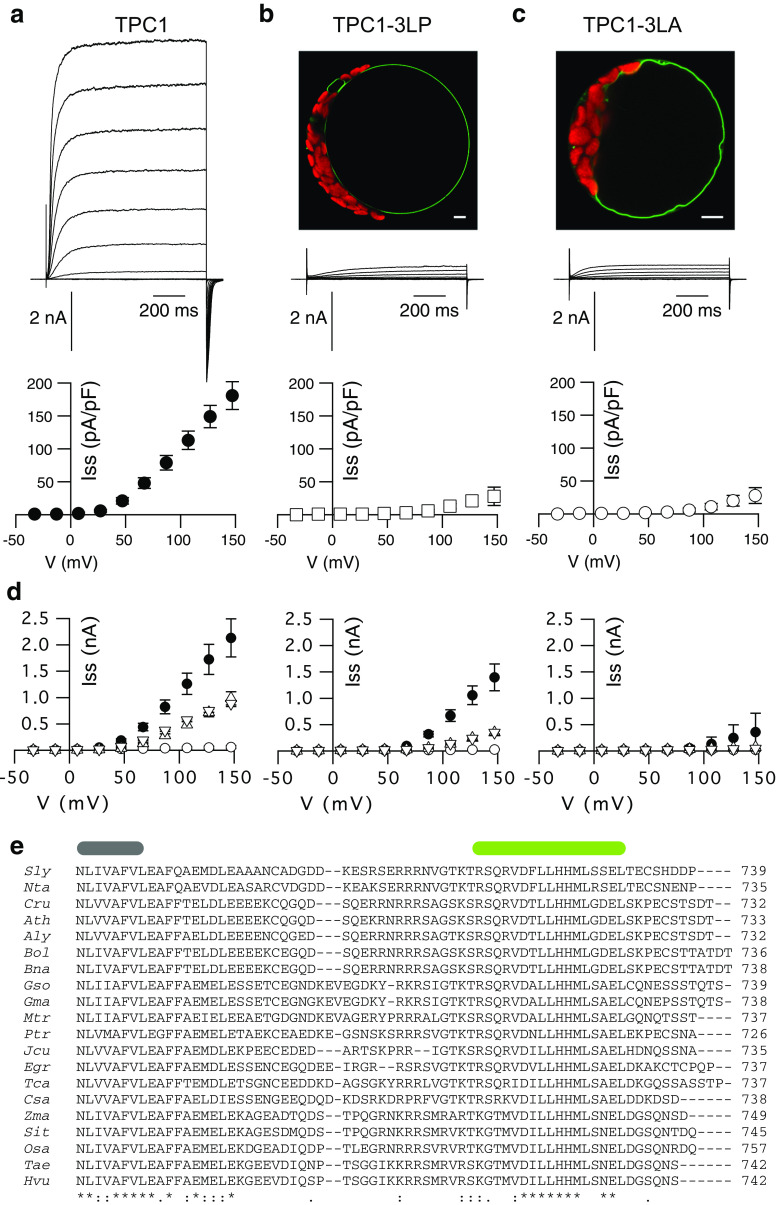
Point mutations within a conserved *C*-terminal region reduce TPC1 activity. **a**–**c** Representative whole-vacuolar current responses (*top*) and current–voltage relations (*bottom*) of corresponding steady-state currents of TPC1-GFP variants as indicated at the *top*. Applied voltages ranged from −73 to +147 mV in 20 mV intervals, starting from a holding potential of −53 mV; current–voltage *curves* are shown starting from −43 mV. Additionally shown in **b** and **c** are confocal fluorescence overlay images of the GFP (*green*) and chlorophyll (*red*) signals of corresponding mesophyll protoplasts (*scales* represent 5 μm). **a** TPC1-GFP as in Fig. 1 (*closed circles*, *n* = 4), **b** TPC1-3LP-GFP (*open squares*, *n* = 7), **c** TPC1-3LA-GFP (*open circles*, *n* = 8). **d** Current–voltage relations of excised cytosolic side-out vacuolar membrane patches of TPC1-GFP (*closed circles*), TPC1-S706A-GFP (*upward triangle*), and TPC1-S706D-GFP (*downward triangle*) expressing cells as well as *tpc1*-*2* mutants (*open circles*), with cytosolic [Ca^2+^] of 1 mM (*left*), 0.2 mM (*middle*), or 0.05 mM (*right*), respectively (*n* = 3–7). **e** *C*-terminal alignment of TPC1 from different plant species.* Gray bar* indicates the end of transmembrane segment S12, as predicted for AtTPC1 [84]. *Green bar* denotes the predicted *C*-terminal helix for AtTPC1. *Aly*-*Arabidopsis lyrata*; *Ath*-*Arabidopsis thaliana*; *Bna*-*Brassica napus*; *Bol*-*Brassica oleracea*; *Cru*-*Capsella rubella*; *Csa*-*Cucumis sativus*; *Egr*-*Eucalyptus grandis*; *Gma*-*Glycine max*; *Gso*-*Glycine soja*; *Hvu*-*Hordeum vulgare*; *Jcu*-*Jatropha curcas*; *Mtr*-*Medicago truncatula*; *Nta*-*Nicotiana tabacum*; *Osa*-*Oryza sativa*; *Ptr*-*Populus trichocarpa*; *Sit*-*Setaria italica*; *Sly*-*Solanum lycopersicum*; *Tae*-*Triticum aestivum*; *Tca*-*Theobroma cacao*; *Zma*-*Zea mays*

To assess the role of this α-helix we investigated a mutant with an altered secondary structure. Substitution of three leucines (L714, 715, and 719) by prolines as a potent helix-breaker [65] resulted in the mutant TPC1-3LP, which, according to the secondary structure prediction tools listed in Table 1, lacks the α-helix. In a complementary experiment, a second point mutant, TPC1-3LA, was chosen to replace bulky hydrophobic leucines by the comparably small alanine favoring an α-helical conformation [66]. The prediction tools as listed in Table 1 supported the assumption of a helical secondary structure in the *C*-terminus of TPC1-3LA. The fluorescence emerging from the GFP-tagged mutant was detected in the tonoplast of transformed cells, but whole-vacuolar currents of TPC1-3LP produced only about 7 % of the wild type amplitude (Fig. 2a, b). Interestingly, TPC1-3LA resulted in a loss of SV currents to a similar extent as TPC1-3LP (Fig. 2c). Comparable expression levels between wild type and mutants were indicated by similar GFP intensities, both on the single cell level and for the protoplast suspensions (Fig. 2b–c, Fig. S1). Residual currents produced by both mutants displayed the typical voltage- and time-dependent characteristics of the SV channel. In excised patches, no macroscopic currents were resolved, but the presence of single-channels with a conductance of 58 pS, similar to that of the wild type [14, 20, 67], showed that the single channel amplitudes were not affected by the mutation (Fig. S2).

A current reduction was also obtained, when a point mutation was introduced at Ser706, which is located at the *N*-terminal end of the helix (Table 1). Since Ser706 represents a putative phosphorylation site, it was replaced either by the phosphorylation-mimicking Asp, introducing a negative charge, or by Ala. In both cases, the whole-vacuolar current amplitudes were largely reduced, by 58 % and 53 % for TPC1-S706D and TPC1-S706A, respectively (Fig. 2d, Fig. S3). The current reductions were also observed at non-saturating Ca^2+^-concentrations of 0.2 mM and 0.05 mM (Fig. 2d). Similar open probabilities of wild type and mutants at saturating Ca^2+^ concentrations (1 mM) and 0.2 mM, corresponding to the half-maximal activation concentration [13], showed that the current reductions did not result from a largely impaired Ca^2+^-dependent shift of the voltage dependence (Fig. S3). Besides shifting the voltage dependence to less negative potentials, an elevation of the cytosolic Ca^2+^ concentration also increases the maximum conductance [13]. While TPC1-S706 wild type currents were reduced to 53 ± 10 % at 107 mV following the reduction of Ca^2+^ from 1 mM to 200 µM, this value was 28 ± 3 % for TPC1-S706A and 24 ± 4 % for TPC1-S706D (Fig. 2d). The S706 mutation therefore appears to modestly affect the Ca^2+^-sensitivity of channels via the link between the Ca^2+^-binding and change in the number of voltage-sensitive channels.

Together, these results show that Ser706 appears to have a structural role instead of being involved in channel regulation by phosphorylation. Furthermore we conclude that mutations in or near the *C*-terminal helix reduce the number of open channels rather than affecting the voltage dependence itself.

An alignment of the TPC1 carboxyl-termini of different species shows that the *C*-terminal helix (CTH), including S706 as well as L714, L715, and L719, is highly conserved among plants (Fig. 2e), implicating an important function of this domain. Our results strongly suggest that vacuoles harboring mutations in the *C*-terminal helix formed less functional two-pore channels. As helical structures are often involved in mediating protein–protein interactions, the CTH of TPC1 may be involved in protein–protein interactions required as a prerequisite for channel gating or stabilization of the open state.

### TPC1 dimerizes via its *C*-termini

One possibility for a CTH-mediated interaction would be a dimerization of the cytosolic *C*-termini of two TPC1 subunits. To test this possibility, interaction studies were performed using bimolecular fluorescence complementation (BiFC) and co-immunoprecipitation (Co-IP).

**Fig. 3:**
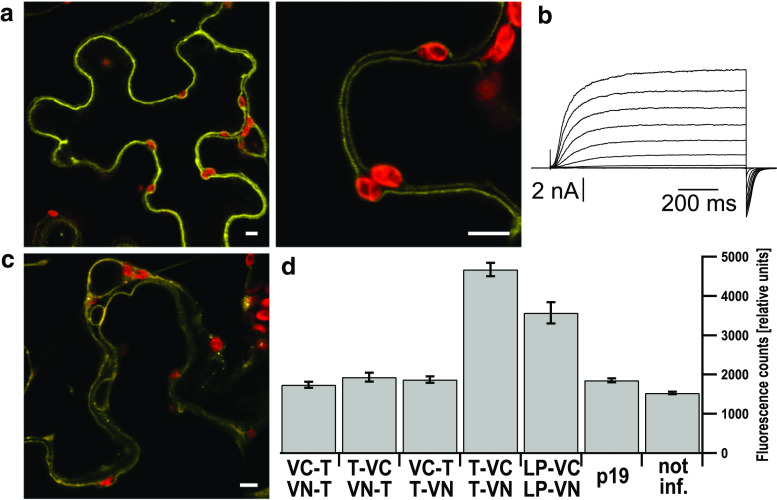
The *C*-termini of two TPC1 monomers interact with each other. **a** Confocal fluorescence overlay images of the Venus (*yellow*) and chlorophyll (*red*) signals of a tobacco cell co-expressing TPC1 fused to Venus-Ct (TPC1-VC) and TPC1 fused to Venus-Nt (TPC1-VN), shown at two different magnifications (*scales* represent 5 μm). The BiFC signals emerge from the tonoplast. **b** Whole-vacuolar current response of a *tpc1*-*2* cell co-expressing the same constructs as in (**a**). **c** Confocal fluorescence overlay image of the YFP (*yellow*) and chlorophyll (*red*) signals of a tobacco cell co-expressing TPC1-3LP-VC and TPC1-3LP-VN (scales represent 5 μm). The BiFC signals emerge from endomembranes and tonoplast. **d** Fluorescence signal intensities of BiFC experiments in relative units, determined from leaf disks with a fluorescence reader. VC-TPC1 and VN-TPC1 (*n* = 23), TPC1-VC and VN-TPC1 (*n* = 23), VC-TPC1 and TPC1-VN (*n* = 23), TPC1-VC and TPC1-VN (*n* = 38), TPC1-3LP-VC and TPC1-3LP-VN (*n* = 11). As negative controls leaf disks only expressing the helper strain p19 (*n* = 28) and not infiltrated leaves (*n* = 39) were used

Split Venus BiFC was tested for the full-length channels, either the wild type TPC1 or the TPC1-3LP mutant. BiFC signals from wild type channels could be observed when two *C*-terminally tagged TPC1 subunits were co-infiltrated in *Nicotiana benthamiana* (Fig. 3a). The fluorescence signals emerged from the vacuolar membrane and in few cells additionally from the preceding endomembranes of the secretory pathway, such as endoplasmic reticulum or Golgi apparatus. This shows that the *C*-terminal parts of TPC1 come into close contact to one another, allowing for fluorophore formation. Patch-clamp analysis of *tpc1*-*2* *Arabidopsis* cells co-transformed with the *C*-terminally tagged TPC1 BiFC-constructs resolved typical SV currents, supporting the hypothesis that a dimerization via the *C*-termini results in voltage-sensitive channels (Fig. 3b).

TPC1-3LP *C*-terminally tagged with the Venus halves produced BiFC signals that were reduced compared to the wild type (Fig. 3d). Confocal fluorescence microscopy revealed that compared to the wild type, TPC1-3LP tagged with the Venus halves was less efficiently transported to the vacuole (Fig. 3c), since the fluorescence emerged not only from the vacuolar membrane, but also to significant amounts from other endomembranes, mostly the ER. This localization pattern of TPC1-3LP with BiFC was not seen with GFP-tagged TPC1-3LP in *Arabidopsis* *tpc1*-*2* cells (Fig. 2b, Fig. S1) and might hint to a slowed trafficking associated with the tobacco expression system compared to the *Arabidopsis* mesophyll protoplasts. Nevertheless, the reduced BiFC signals of the helix-breaking point mutant TPC1-3LP (Fig. 3c) in comparison to the wild type may indicate that the dimer formation via the *C*-terminus was hampered. However, the flexibility of the *C*-termini and Venus halves would still allow the formation of the fluorophore.

In contrast, combinations with *N*-terminally tagged TPC1 subunits did not result in Venus-fluorescence making intramolecular interactions of the *N*-terminus seem unlikely (Fig. 3d). Functional expression and tonoplast localization of an *N*-terminally tagged TPC1 were verified using a GFP tag (Fig. S4).

**Fig. 4:**
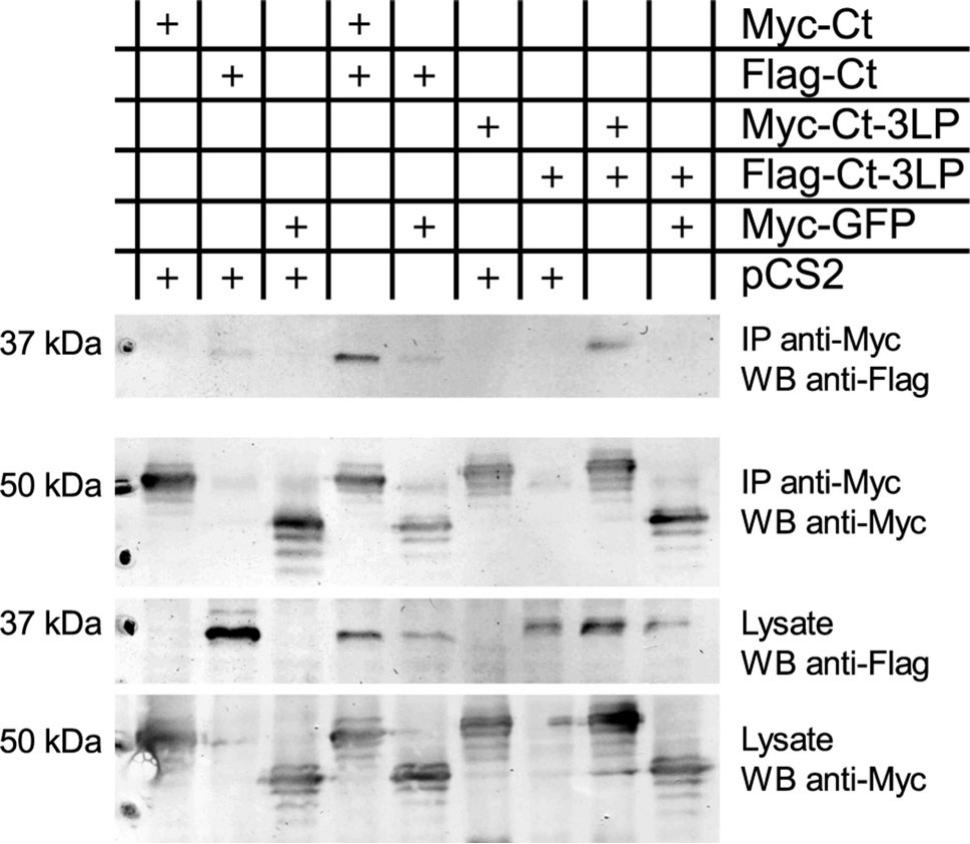
The *C*-terminus of TPC1 interacts with itself. Co-immunoprecipitation of TPC1 *C*-terminal proteins co-expressed in HEK 293T cells (combinations as indicated) and analyzed by western blotting. Precipitation was achieved via the Myc-tag (IP anti-Myc), and proteins detected via antibodies against the Myc- (WB anti-Myc) or the Flag-tag (WB anti-Flag). The Flag-tagged *C*-terminus of TPC1 (Flag-Ct) was co-precipitated with the Myc-tagged *C*-terminus of TPC1 (Myc-Ct, lane 4), whereas only background interaction was observed with Myc-tagged GFP (lanes 2 and 5). Introduction of the 3LP mutation resulted in visibly weaker interaction of the two *C*-termini (lane 8). Similar results for the interaction of the TPC1 *C*-termini were obtained in 3 independent experiments. The presence of the proteins was verified in the cell lysate. Calculated protein weights were Myc-Ct (46 kDa), Flag-Ct (37 kDa), 3LP-Myc-Ct (47 kDa), 3LP-Flag-Ct (38 kDa), Myc-eGFP (37 kDa), Myc (10 kDa), Flag (1 kDa)

In an independent approach, the soluble *C*-termini (residues 673–732) of wild type TPC1 and of TPC1-3LP were assayed for co-immunoprecipitation in a heterologous system. The proteins were *N*-terminally tagged with a 6xMyc-tag for precipitation or a FLAG-tag for detection of co-precipitated proteins, and in addition *C*-terminally enlarged by a GFP-fusion to provide better handling, and co-expressed in HEK293T cells. These experiments confirmed that the TPC1 *C*-terminus bound to itself and could be detected with both antibodies in the immuno-precipitate (Fig. 4). As a control, the empty vector pCS2 or with 6xMyc-GFP was co-transfected with the Flag- or Myc-tagged *C*-terminus, respectively, since GFP is known to have a tendency to dimerize itself. Compared to the wild type, the *C*-terminus of the TPC1-3LP-mutant showed a reduced ability to dimerize, reflected by a weaker signal from the Co-IP, while the expression level was comparable to the wild type (Fig. 4).

**Fig. 5:**
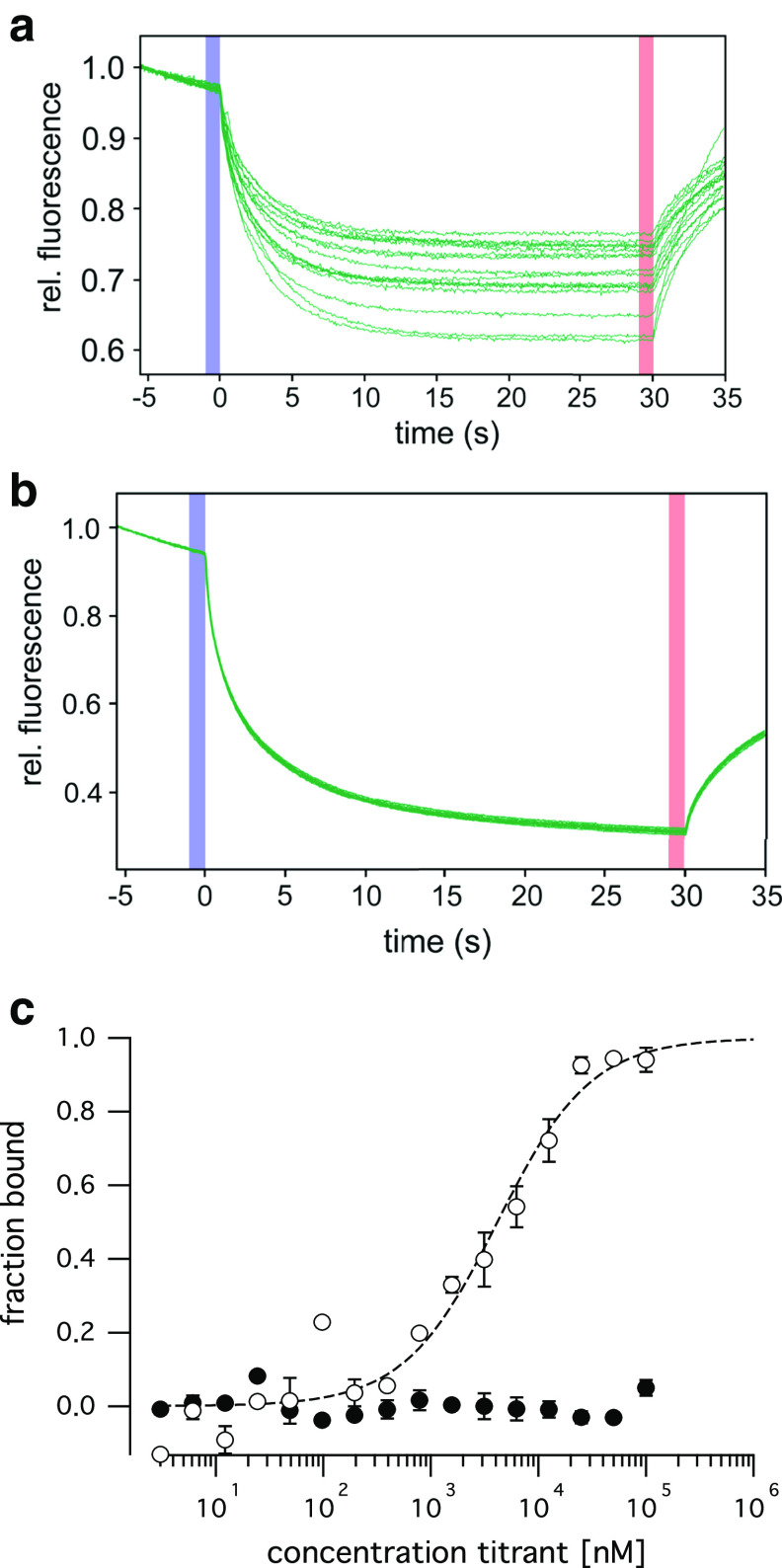
Synthetic CTH peptides dimerize. **a**, **b** MST time traces (normalized fluorescence) of 16 capillaries containing 750 nM tryptophane-labeled wild type (**a**) or 3LA mutant (**b**) CTH peptide and unlabeled wild type (**a**) or 3LA mutant (**b**) CTH peptide at concentrations between 3 nM and 100 µM. Thermodiffusion is reduced with increasing peptide concentrations. **c** The normalized fluorescence of the MST traces was converted to the fraction bound and plotted against the concentration of the ligand. A *K*_D_ of 3.85 ± 1.1 µM was determined for the interaction of the wild type CTH peptides (*open circles*), while no interaction was measured for the 3LA mutant (*closed circles*)

The two independent protein–protein interaction assays demonstrated the dimerization of the TPC1 *C*-terminus and suggest that this interaction is mediated by the *C*-terminal α-helix (CTH). The ability of the CTH to homo-dimerize was further directly measured in microscale thermophoresis (MST) experiments. Titration of the wild type peptide corresponding to the CTH (Table 1) against the wild type peptide labeled by tryptophane induced a dose-dependent change in mobility (Fig. 5a), indicative of the dimer formation. From the change in fluorescence a binding affinity of 3.85 ± 1.1 µM was determined (Fig. 5c). In contrast, titration curves of the 3-LA peptide revealed no interactions of this mutant peptide in a concentration range from 3 nM to 100 µM (Fig. 5b,c).

### The CTH mediates formation of an antiparallel coiled-coil dimer

The CTH-mediated dimerization was further analyzed in silico, both for the wild type and for the CTH-3LA mutant. To this end, the dimerization was first analyzed from 100 aggregation simulations (250 ns each) for each system. Each dimerization simulation started from two *C*-terminal TPC α-helices at an initial peptide center of mass (COM) distance between 5.5 and 6.5 nm solvated in a box of water at coarse-grained resolution. Dimers were formed for both systems in all simulations within tens of nanoseconds, reflecting the observed enhanced stickiness of the coarse-grained MARTINI force field [47].

**Fig. 6:**
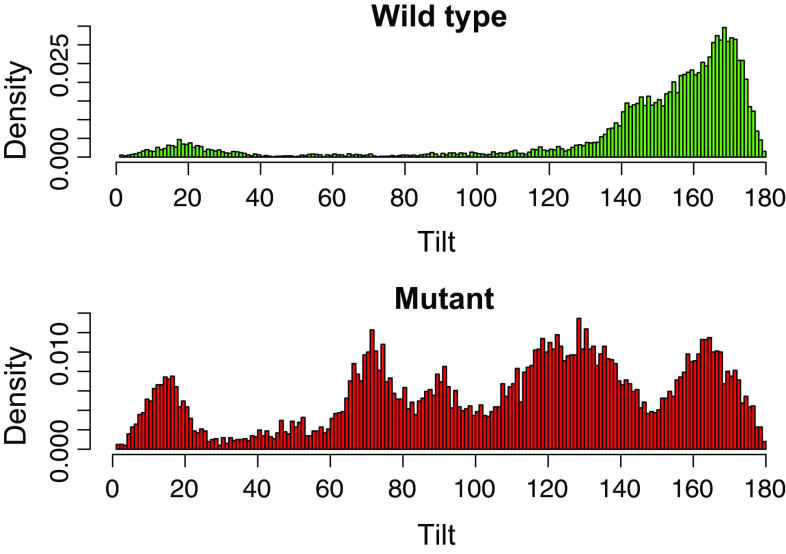
CTH dimer tilt distribution reflects an increased flexibility of mutant dimers. Tilt angles between coarse-grained dimers were determined over the last 50 ns from each simulation and plotted in a *histogram* (*x*-axis: tilt, *y*-axis: density). The density distribution of tilt angles between mutant dimers (*bottom*) is compared to wild type dimers (*top*) very diffuse, pointing to an increased flexibility of mutant dimers

**Fig. 7:**
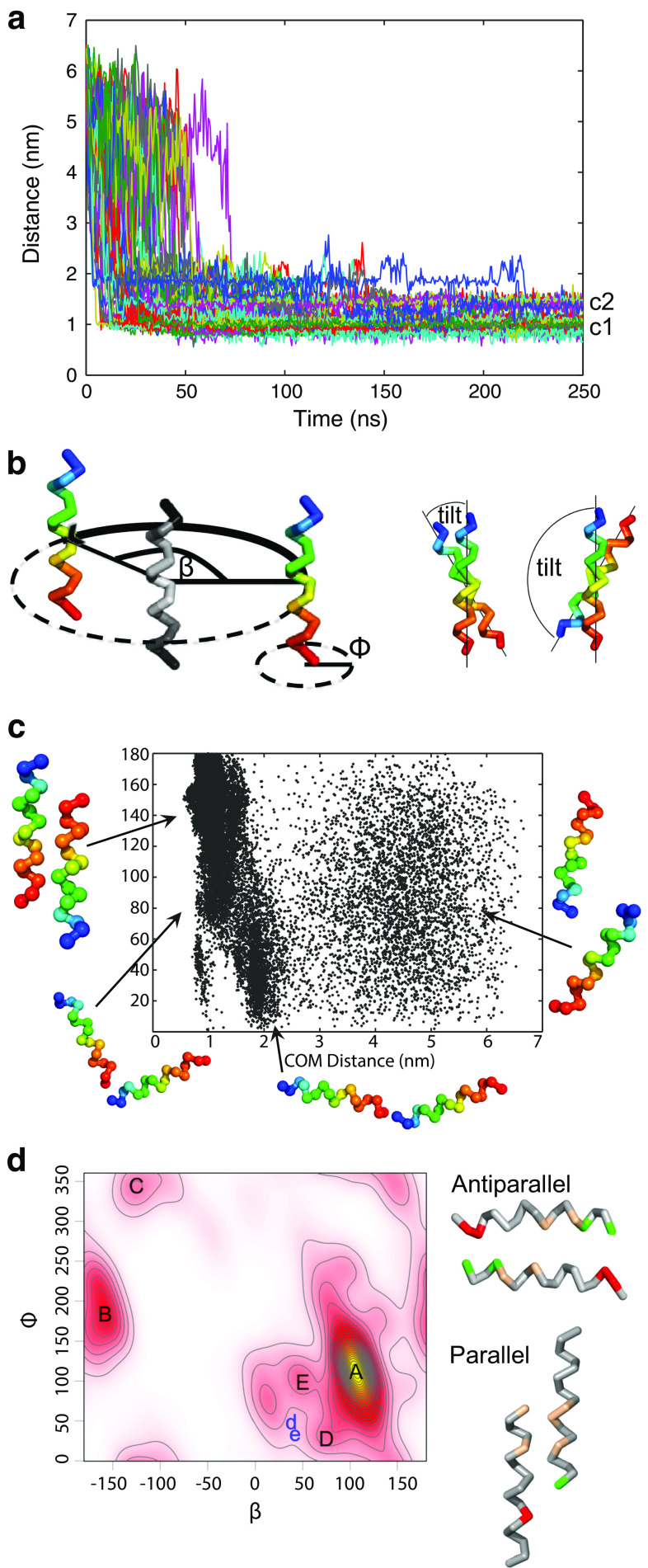
In silico dimerization study of the carboxy-terminus of TPC1. **a** Center of mass (COM) distance between the two helices (residues 707–723), with initial values between 5.5 and 6.5 nm, as a function of simulation time. Different colored lines represent 100 independent coarse-grained (CG) simulations. c2 and c1 indicate two populations of dimers distinguished by their COM distance. **b** Schematic representation of angles used to analyze the orientation of bound helices. *β* describes the position of the binding partner to the reference structure (*colored in gray*), *ϕ* is the rotation around the helical axis, and the tilt angle depicts the tilt between the peptides. Lower tilt values (≤50°) point to parallel binding and larger (≥130°) to antiparallel binding. Helices were colored in rainbow scheme from *N*-terminus (*blue*) to *C*-terminus (*red*). **c** Center of mass distances of two monomers forming homo-dimers in antiparallel arrangement, shown for all simulations resulting in antiparallel configurations. **d** *Left*: Kernel density for *β* and *ϕ* angle combinations in the last 50 ns of the CG simulations, describing the relative orientation of peptides. The *yellow* *area* indicates a high density, *white color* a low density. Antiparallel dimers were found in clusters A and B, parallel dimers in C and D, others in E. Two selected conformations from clusters D and E were finally observed at positions in phase space d and e, respectively, after backmapping to atomistic resolution followed by atomic-scale molecular dynamics simulations. *Right* Orientation for the antiparallel and parallel homo-dimers. Glutamic and aspartic acids are colored *red*, arginine *green*, and hydrophobic residues *light brown*. For clarity, only interacting residues are colored

However, the dimerized CTH-3LA adopted significantly different relative orientations as compared to wild type dimers: Overall, the mutant exhibited more flexibility in the bound state (Fig. 6, Fig. S5). The angle between the helical axes, called tilt, describes the parallel or antiparallel orientation of the bound peptides (Fig. 7b). The distribution of this angle (Fig. 6), calculated over the last 50 ns of the trajectories, is in the case of the CTH-3LA dimers very diffuse, pointing to a drastically enhanced flexibility and thus less stable dimers as compared to the wild type dimers. The number of transitions between the orientations (see “Data Analysis”) strengthens this conclusion: While the majority of wild type dimers were stable in their orientation (median of orientation transitions is 0), the number of transitions in mutant dimers was significantly increased (median is 4.5, Fig. S6). This flexibility in the relative orientation of mutated helices most likely results from a reduction in steric hindrance. Leucines consist of two coarse-grained beads (four residual methyl groups), while alanine exhibits only one backbone bead (one residual methyl group). Since the majority of wild type dimers ended up in a conformation, where leucines are buried in the interface (see below), this exchange to the smaller alanines caused an increased flexibility in the tilt angle. Furthermore, alanines are not as hydrophobic as leucines and thus, they are more prone to face the solvent.

The relative instability and promiscuity of the CTH-3LA dimers observed in MD simulations corresponds to the lack of dimer formation in MST experiments. In the following, we focused on the analysis of dimer formation for the CTH wild type peptide.

Within approximately 50 ns, the wild type peptides approached each other in all simulations to a distance below 1 nm, reflecting dimerization (81 % of the monomers dimerized within 25 ns). Figure 7a shows the center of mass distances for each simulation. With increasing simulation time, cluster formation is seen, with one cluster at a COM distance of ≈1 nm (c1) and a second cluster at ≈1.45 nm (c2). Peptides in dimers of cluster c2 adopted a shifted configuration (see below).

The orientation of the *C*-terminal dimers of TPC1 was analyzed using Euler angles [47, 64]. A tilt angle below 50° indicates that the helices are in a parallel conformation, while an angle above 130° reflects an antiparallel orientation (Fig. 7b). The latter configuration was adopted in 86 % of the coarse-grained simulations, characterized by an average tilt angle of 160°. In 7 % of the simulations, the dimers ended up in a parallel orientation (average tilt of 18°), the remaining simulations ended up in diverse intermediate configurations. To reach the antiparallel conformation most of the dimers followed a specific dimerization pathway (Fig. 7c): At larger intermolecular distances (3–7 nm), i.e., at the beginning of the simulation, the distribution of tilt angles was random (Fig. 7c). Upon approach, the tilt was constrained to values of 10–50°, the peptides thus oriented in a parallel head-to-tail configuration. Subsequently, the peptides reoriented and ended up in the antiparallel configuration (130°–180°, Fig. 7c).

To evaluate the frequency of the different dimer conformations at the end of the simulations, combinations of the binding position *β* and rotation angle *ϕ* (compare Fig. 7b) obtained from the trajectories were plotted as a two-dimensional kernel density map (Fig. 7d). While the position of one monomer to a reference monomer is defined by the position *β*, the exact binding site that faces the reference structure is given by the phase *ϕ*, the rotation around its helical axis (Fig. 7b).

In the bound state, i.e., at the end of the simulations, positively and negatively charged amino acids come into close proximity, in particular in the antiparallel configuration (Fig. 7d). These antiparallel dimers (cluster A in Fig. 7d) are electrostatically favored by proximity of the *N*-terminal arginines of one monomer and the *C*-terminal glutamic and aspartic acids of the second monomer. Additionally, the hydrophobic residues leucine and valine are positioned at the dimer interface.

Besides the main cluster A, four additional clusters (B–E) were identified. The second-largest cluster B consists of antiparallel dimers that interact as well through electrostatic interactions. Due to a decreased number of hydrophobic residues at the dimer interface, this conformation is expected to be metastable. Parallel dimers were found in clusters C and D. In these arrangements, the helices are shifted with respect to each other, allowing for a better packing of hydrophobic residues (parallel dimer, Fig. 7d). Therefore, the center of mass distances between the corresponding monomers is increased as compared to the antiparallel dimers (compare c2 in Fig. 7a). For cluster E (tilt from 50°–130°) the inner leucines are in close proximity to each other. Furthermore, in this configuration the central aspartic acids are able to interact with the arginines.

To evaluate the binding strength, the non-bonded interaction energy was calculated for the different conformations. In agreement with the high frequency of antiparallel dimers (cluster A and B), their mean interaction energy was lowest among all dimers with a value of ≈−600 kJ/mol, while the parallel dimers interact with ≈−420 kJ/mol, and the rest found in cluster E with ≈−500 kJ/mol.

**Fig. 8:**
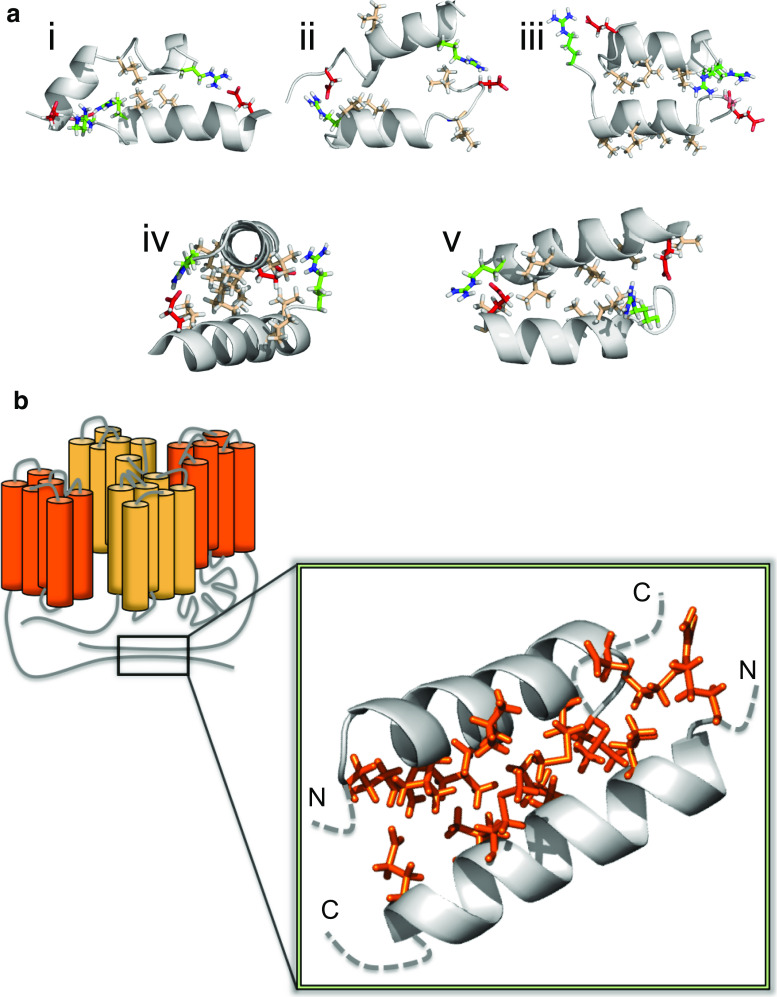
CTH dimer configurations observed in combined coarse-grained/atomistic simulations. **a** Metastable (*i*, *ii*, *iii*) and stable (*iv*, *v*) dimer configurations. For the stable dimer arrangement, the hydrophobic residues are found mainly at the interfacial region, and stabilizing salt bridges at the termini. For clarity, interfacial methionines are hidden. The less stable configurations show partially exposed hydrophobic (*light brown*) amino acids and a disrupted helical conformation. Glutamic and aspartic acids are colored *red* and arginine *green*. **b** Model for the functional TPC1 with the antiparallel CTH dimer enlarged (Shaker domains I and II are differently colored in each monomer). A head-to-tail conformation is assumed for the TPC1 dimer, as suggested by Rietdorf et al. [10]

For further analysis of the stability of the dimers formed at coarse-grained (CG) resolution, five CG conformations corresponding to the main clusters A–E as indicated in Fig. 7d were backmapped to atomistic resolution and simulated for 200 ns (cluster A, C–E) or 270 ns (cluster B). The two initial parallel dimer configurations (C and D) changed in the atomistic simulations to an antiparallel orientation within less than 10 ns, indicating a relative instability of the parallel configurations. The dimers of clusters D and E adopted an antiparallel configuration (position *d*, *e* in Fig. 7d) during the atomistic simulations, with the three inner leucines and valine at the helical interface stabilized by two salt bridges at the termini (Fig. 8a, iv and v). This orientation is indicative of coiled-coil packing, burying hydrophobic residues within the core flanked by stabilizing ionic interactions at the helical sides.

Less stable conformations were observed for the other dimers (clusters A, B, C). Here, leucines and valines were partially exposed to the solvent (Fig. 8a, ii and iii) or hydrogen bonds within the helix were broken and consequently the helix disrupted (Fig. 8a, i and ii). Additionally, hydrophilic histidines were partially found at the interface.

The combined coarse-grained and atomistic study thus strongly suggests that the wild type, but not the mutant CTHs of two TPC1 monomers form a stable helical dimer in an antiparallel coiled-coil conformation (Fig. 8b). Together with the results of the mutagenesis study, we propose the formation of an antiparallel CTH dimer as a prerequisite for TPC1 activity. This conclusion is further supported by the ability of the synthetic CTH peptide to rapidly inhibit TPC1 currents, when applied to the active channels in electrophysiological recordings (Fig. S7).

## Discussion

Here, we identified the presence of a carboxy-terminal helix (CTH) in TPC1 and documented its role for channel dimerization and function. Loss of the CTH rendered the channel inactive while point mutations within the CTH resulted in severely reduced channel activity. Correspondingly, BiFC analysis and Co-IP experiments reported on the interaction of the *C*-terminus, which was reduced by mutations within the CTH. A residual current of 10 % in the TPC1-3LA and -3LP mutant may indicate that in contrast to the CTH peptide the complete *C*-terminus may dimerize to a small extent even with three leucines replaced. Although we cannot exclude the possibility that in addition to the CTH other parts of the *C*-terminus are involved in the dimerization process, the lack of the CTH was sufficient to render the channel silent.

Dimerization of the CTH was directly shown in MST assays and MD simulations. The sequential multiscale simulations revealed that the wild type CTH dimer preferably adopts an antiparallel coiled-coil conformation. This antiparallel orientation is in accordance with an assumed head-to-tail configuration of the channels [10]. The fact that leucine to alanine mutations in the inner helix drastically impaired channel function is in accordance with the observation that although alanine is a residue of high helix propensity, it can decrease the stability of the helix (dimer) [66, 68]. Indeed, coarse-grained MD simulations discovered mutated dimers that exhibit a significant increase of flexibility, pointing to less stable dimers. These results were corroborated by MST analyses, which showed that the wild type CTH dimerized with a *K*_d_ of 3.9 µM while the mutants did not interact. Apparently, the CTH is not required for correct assembly of the channel in the membrane. However, our results suggest that the CTH is crucial for stabilization of the TPC1 dimer and coupled with dimerization also for channel gating.

Gating of ion channels by their soluble termini is a common feature of ion channels [69–73], and often modification of the *C*-terminus induces alterations of the voltage dependence [74–77]. In comparison, mutations within and near the CTH did not feedback onto the voltage dependence of TPC1. In this respect, TPC1 shares structural and functional properties with voltage-dependent sodium (Na_V_) channels. Deletion of the complete or distal part of the *C*-terminus from a prokaryotic Na_V_ resulted in a complete or almost complete loss of sodium currents, respectively, while the trafficking of the channel and the voltage dependence of activation were unaffected [70]. Combined MD simulations and electron paramagnetic resonance spectroscopy showed that a *C*-terminal helix of this channel forms a coiled-coil bundle involving four subunits, and that this tetramerization is essential for coupling to channel opening via a proximal *C*-terminal linker following S6. Most notably, the linker contains a negatively charged cluster, and several glutamate residues are also conserved in the plant TPC *C*-termini following S6 (Fig. 2). A role of the coiled-coil in stabilizing the sodium channel tetramer or dimer in case of TPC1, and in enabling the opening and closing of the pore during gating without disrupting the quaternary structure may thus represent a mechanism also valid for two-pore channels, which has to be further evaluated in future studies.

Besides voltage changes, TPC1 is activated by binding of Ca^2+^ ions to the EF-hands in the central linker domain between transmembrane S6 and S7, which apparently stabilizes the open state [13, 78, 79]. The rabbit skeletal muscle type 1 ryanodine receptor RyR1 is an intracellular Ca^2+^ release channel, which also belongs to the six-transmembrane superfamily. Similar to TPC1, activation of RyR1 by Ca^2+^ involves cytosolic EF-hands, here present in the *N*-terminus, while the *C*-terminus homodimerizes. An essential function of the RyR1 *C*-terminus requires the last 15 amino acids, deletion of which abolishes channel function [80]. Recently, the crystal structure of the RyR1 revealed a putative mechanism for Ca^2+^-mediated gating involving the *C*-terminus [73, 81]. In RyR1 Ca^2+^-dependent changes in the conformation of the *N*-terminal EF-hand containing domain are transmitted to the pore via contacts with the *C*-terminal domain, inducing a change of the cytosolic aperture of the channel and stabilizing the open state [73, 81]. A RyR1-like allosteric mechanism may therefore also account for the role of the *C*-terminus in the Ca^2+^- and voltage-dependent activity of TPC1.

TPC1 is a large-conductance channel and is therefore tightly regulated to prevent ion leakage from the vacuole [reviewed in 2]. The many ionic cytosolic regulators, such as H^+^, Ca^2+^, Mg^2+^, K^+^, and Na^+^, exert their effects via alteration of the voltage dependence of the channel. In contrast, 14-3-3 proteins inhibit TPC1 activity by about 90 % within 10 s without any changes in the voltage dependence [36]. A similar reduction in channel activity without affecting the voltage dependence was observed for the CTH mutants described here. Likewise, application of the synthetic CTH peptide to the vacuolar membrane rapidly inhibited TPC1 currents (Fig. S7). It is therefore tempting to speculate that the 14-3-3 protein GRF6, which regulates TPC1 in *Arabidopsis* [35], interferes with the dimerization of the carboxyl-termini. A region partly overlapping with the CTH contains two serines (S706, S708) and constitutes a putative 14-3-3 binding site [705-KSRSQR, 82]. Accumulation of GRF6 dimers may thus push the CTHs apart, which will destabilize the TPC1 dimer and induce a rapid closure of the channel pore. Alternatively, 14-3-3 proteins may act more indirectly, because a second putative 14-3-3 binding site is predicted to be located in the linker domain between the two EF-hands at T359 [83]. Given a RyR1-like allosteric mechanism, 14-3-3 binding in the linker may interfere with the coordinated mechanism, which links Ca^2+^ binding to the activation of the channel via the *C*-terminal α-helical region. In any case, the coiled-coil mediated dimerization of wild type CTH’s as a prerequisite for channel opening adds a very rapid regulatory mechanism to shut down this large vacuolar conductance, either by destabilizing the TPC1 dimer, or by preventing the allosteric coupling to the channel pore.

### Electronic supplementary material

Below is the link to the electronic supplementary material.

Supplementary material 1 (PDF 14533 kb)

Supplementary material 2 (PDF 840 kb)

Supplementary material 3 (PDF 6444 kb)

Supplementary material 4 (PDF 1385 kb)

Supplementary material 5 (PDF 6799 kb)

Supplementary material 6 (PDF 204 kb)

Supplementary material 7 (PDF 660 kb)

Supplementary material 8 (DOCX 17 kb)
