# Supplementary figures and images for: The function of the two-pore channel TPC1 depends on dimerization of its carboxy-terminal helix
Source: Cell Mol Life Sci. 2016 Jan 18;73(13):2565–81. doi: 10.1007/s00018-016-2131-3 (PMC4894940; doi:10.1007/s00018-016-2131-3)

Figure S1

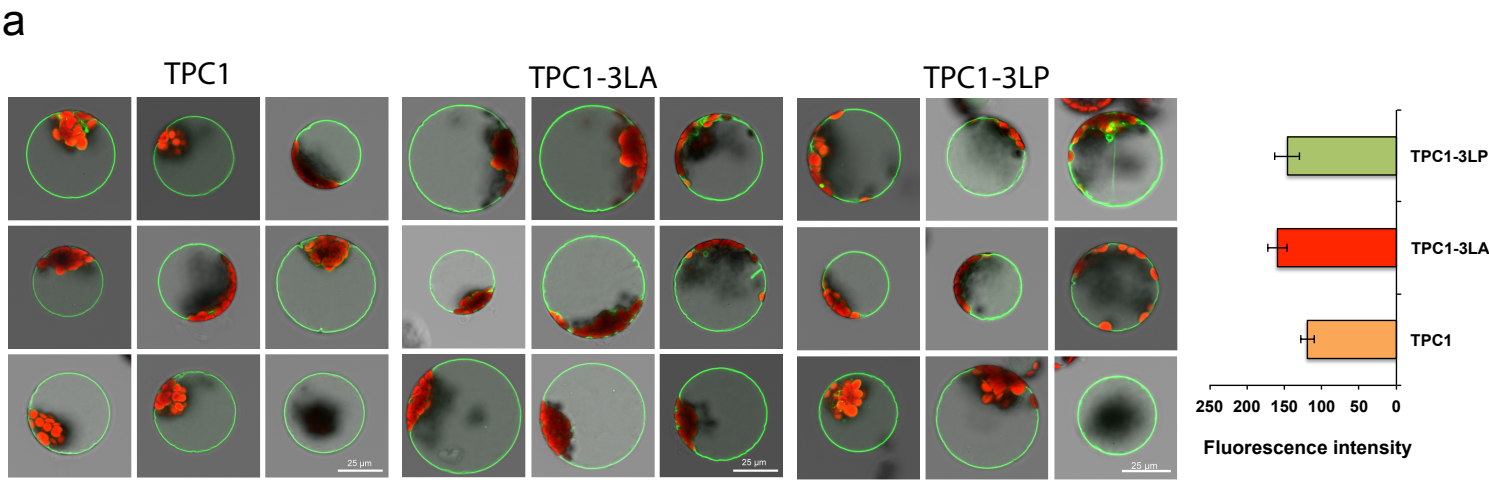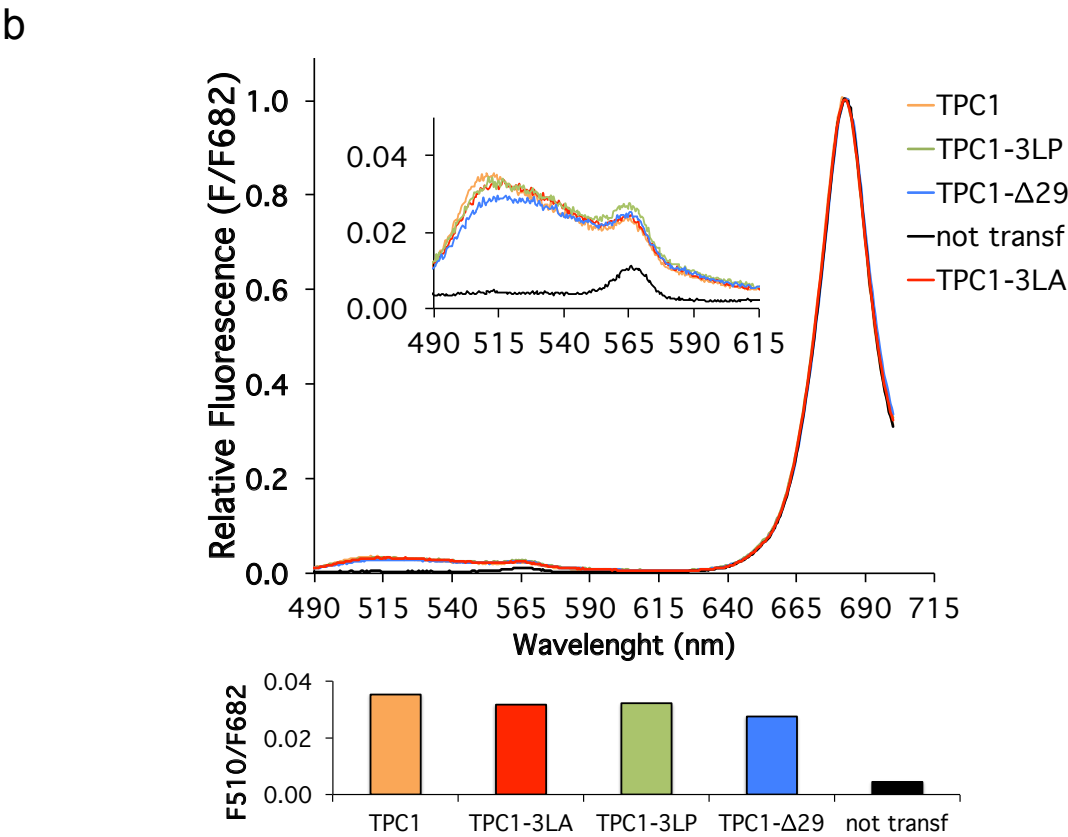

Supplement: Supplementary file 1 — Supplementary material 1 (PDF 14533 kb) [file 18_2016_2131_MOESM1_ESM.pdf]

Figure S2

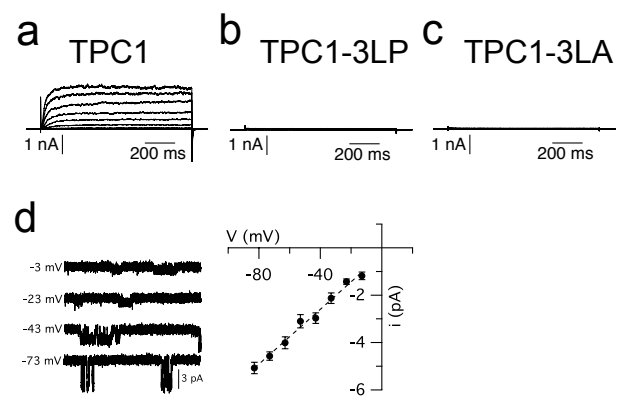

Supplement: Supplementary file 2 — Supplementary material 2 (PDF 840 kb) [file 18_2016_2131_MOESM2_ESM.pdf]

Figure S3

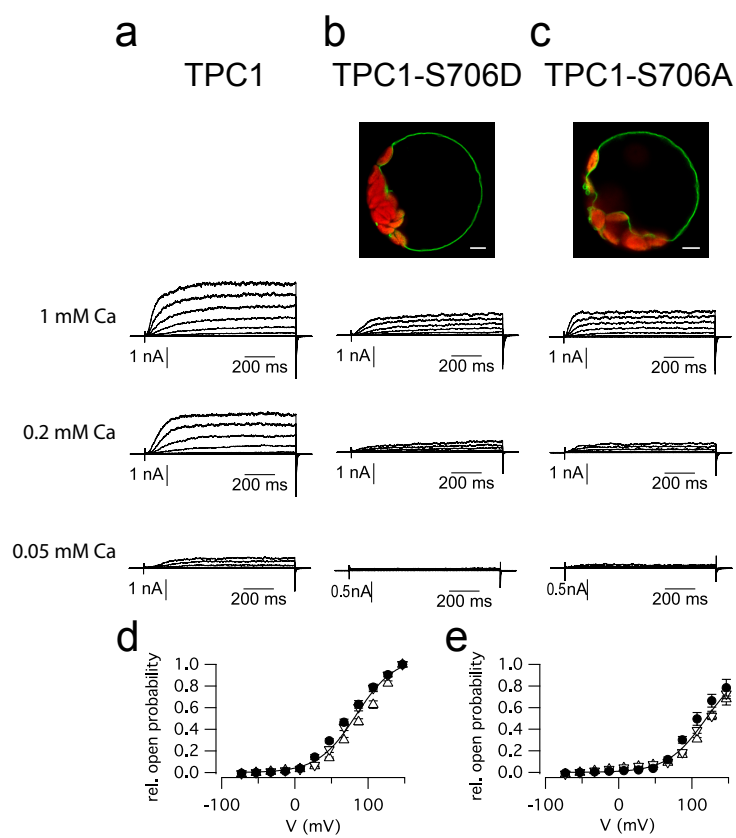

Supplement: Supplementary file 3 — Supplementary material 3 (PDF 6444 kb) [file 18_2016_2131_MOESM3_ESM.pdf]

Figure S4

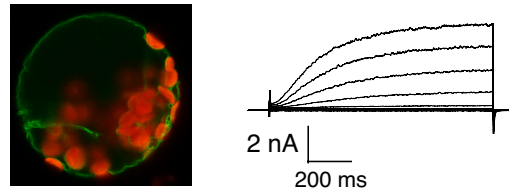

Supplement: Supplementary file 4 — Supplementary material 4 (PDF 1385 kb) [file 18_2016_2131_MOESM4_ESM.pdf]

Figure S5

Wild type

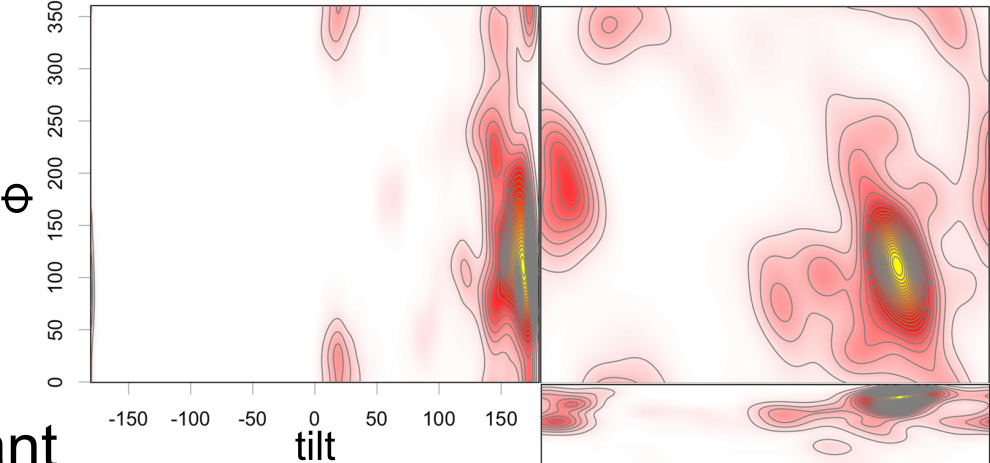

Mutant

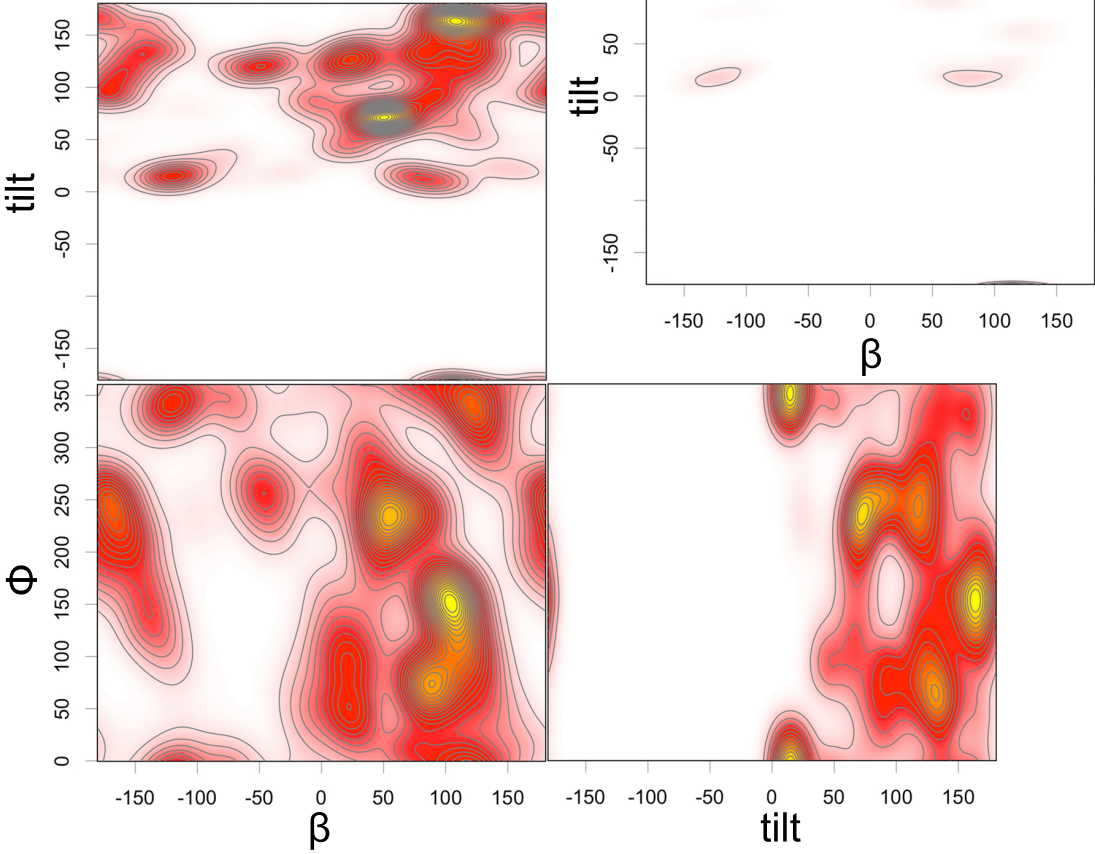

Supplement: Supplementary file 5 — Supplementary material 5 (PDF 6799 kb) [file 18_2016_2131_MOESM5_ESM.pdf]

Figure S6

a

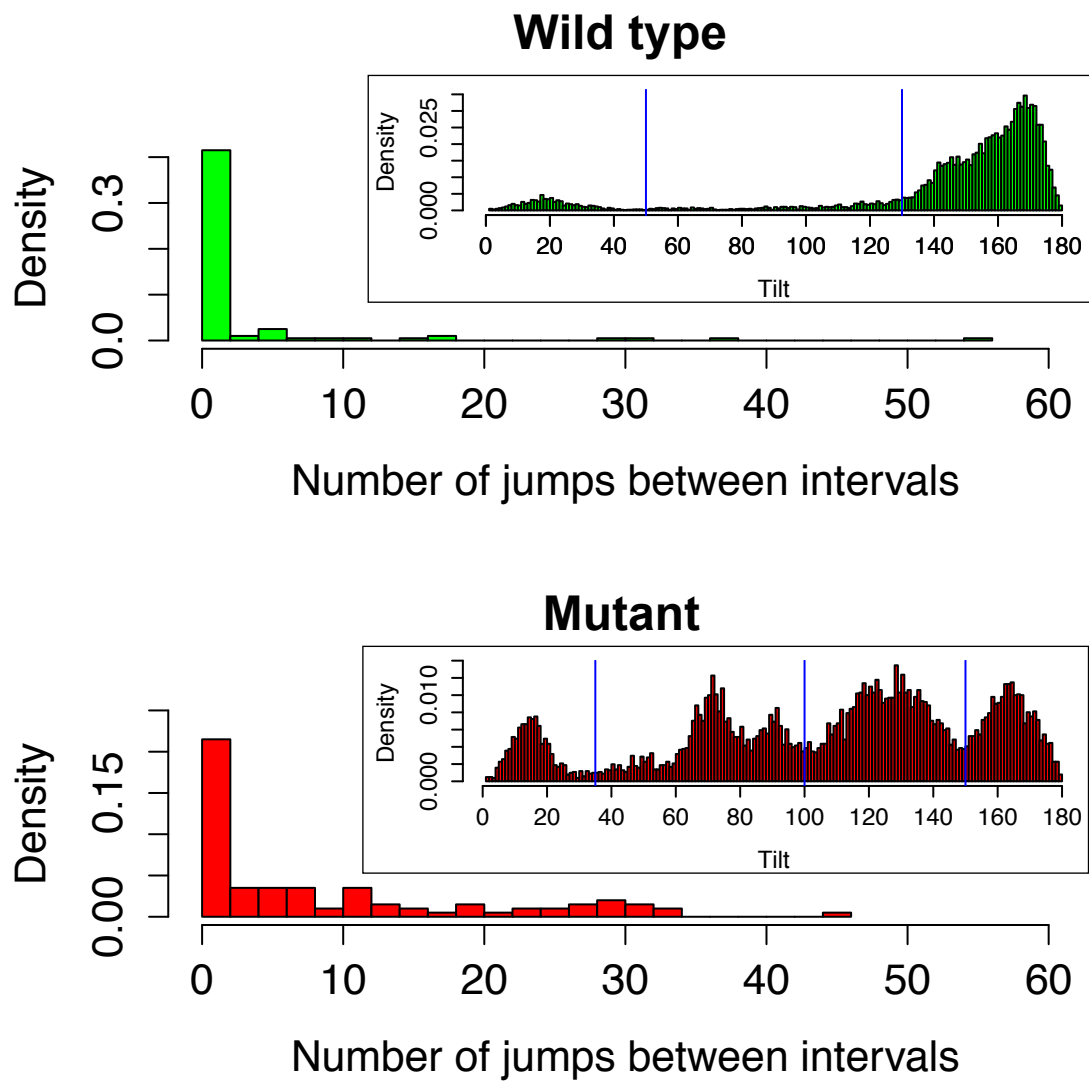

b

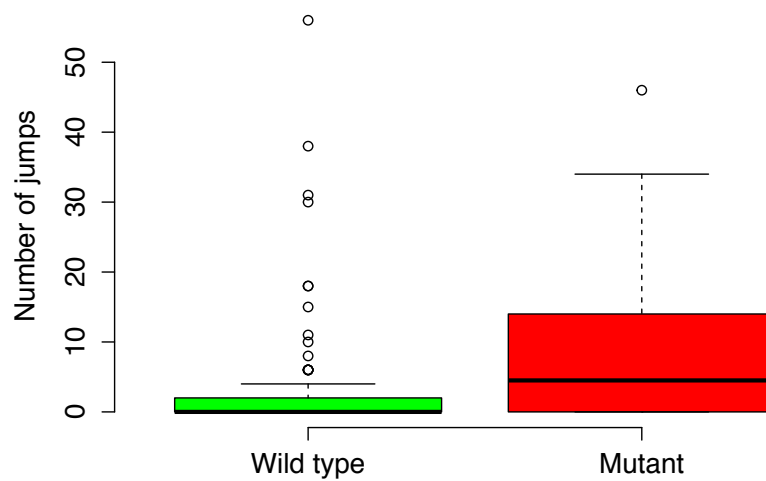

Supplement: Supplementary file 6 — Supplementary material 6 (PDF 204 kb) [file 18_2016_2131_MOESM6_ESM.pdf]

Figure S7

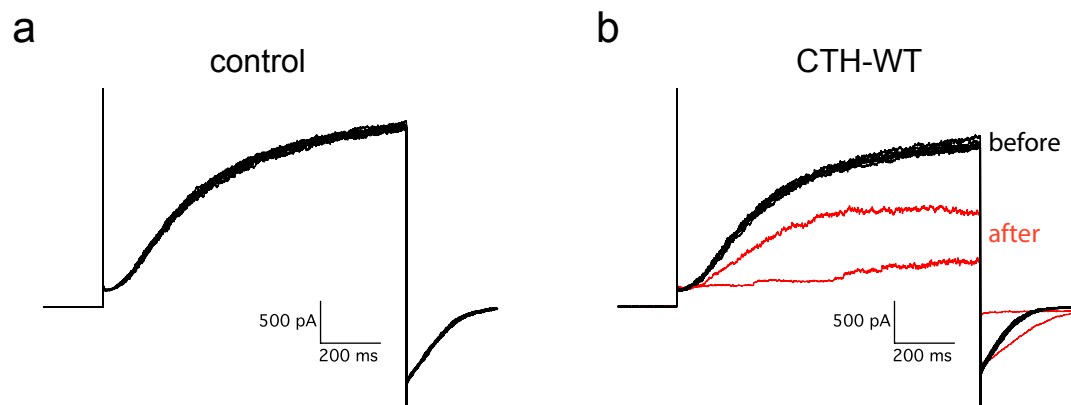

Supplement: Supplementary file 7 — Supplementary material 7 (PDF 660 kb) [file 18_2016_2131_MOESM7_ESM.pdf]
