# Supplementary material for: The function of the two-pore channel TPC1 depends on dimerization of its carboxy-terminal helix
Source: Cell Mol Life Sci. 2016 Jan 18;73(13):2565–81. doi: 10.1007/s00018-016-2131-3 (PMC4894940; doi:10.1007/s00018-016-2131-3)
Supplement: Supplementary file 8 — Supplementary material 8 (DOCX 17 kb) [file 18_2016_2131_MOESM8_ESM.docx]

**Supplemental Figure Legends**

**Figure S1: Quantification of GFP fluorescence from TPC1 fusion proteins.**

**(a)** Confocal fluorescence overlay images of the GFP (green), chlorophyll (red), and bright field images for TPC1 (left), TPC1-3LA (middle), and TPC1-3LP (right). All images were taken at the same magnification and photomultiplier settings and gain for the GFP channel. The mean fluorescence intensity of GFP in the membrane was determined for 10-12 cells for each construct, and shown on the right graph (bars represent standard errors). (b) Fluorescence emission scans for protoplast suspensions transformed with TPC1-GFP (orange), TPC1-3LP-GFP (green), TPC1-3LA-GFP (red), TPC1∆C29 (blue), and non-transformed cells. The fluorescence was expressed relative to the chlorophyll peak at 682 nm to normalize for cell numbers; the inset shows the GFP peaks enlarged. Bars at the bottom are colored as shown in (a) and represent the relative GFP fluorescence intensitiy of the protoplast suspensions at 510 nm, normalized to the chlorophyll fluorescence at 682 nm.

**Figure S2: Analysis of point mutants in the CTH of TPC1 in excised patches.**

**(a-c)** Representative current responses of excised cytosolic side-out patches of *tpc1-2* cells expressing **(a)** TPC1-GFP, **(b)** TPC1-3LP-GFP, **(c)** TPC1-3LA-GFP. **(d)** Single-channel fluctuations in a cytosolic side-out patch excised from a TPC1-3LA-GFP expressing *tpc1-2* cell, and corresponding single channel current-voltage relations. The dotted line represents a linear fit with a slope of 58 pS. Data represent mean values ± SE.

**Figure S3: Mutations of S706 do not alter the voltage-dependence of TPC1.**

**(a-c)** Representative current responses of excised cytosolic side-out patches of *tpc1-2* cells expressing **(a)** TPC1-GFP, **(b)** TPC1-S706D-GFP, **(c)** TPC1-S706A-GFP, in the presence of 1 mM, 0.2 mM, and 0.05 mM Ca^2+^ at the cytosolic side of the membrane. Applied voltages ranged from −73 to +147 mV in 20 mV intervals, starting from a holding potential of −53 mV. Additionally shown in (b) and (c) are confocal fluorescence overlay images of the GFP (green) and chlorophyll (red) signals (scales represent 5 μm) of TPC1-S706D-GFP or TPC1-S706A-GFP expressing mesophyll protoplasts. (**d,e**) Relative open probabilities for TPC1 (open circles), TPC1-S706D (upward triangles), and TPC1-S706A (downward triangles) in the presence of 1 mM (d) or 0.2 mM Ca^2+^ (e) as a function of the voltage, dotted lines represent Boltzmann fits (n=4-7).

**Figure S4: Localization and function of N-terminally GFP-tagged TPC1.**

Confocal fluorescence overlay images of the GFP (green) and chlorophyll (red) signals (scales represent 5 μm), and representative whole-vacuolar current responses of a GFP-TPC1 expressing *tpc1-2* mesophyll protoplast. Applied voltages ranged from −73 to +147 mV in 20 mV intervals, starting from a holding potential of −53 mV.

**Figure S5: Relative orientation of wild type CTH and mutant CTH-3LA.**

Wild type helices form distinct clusters with dimers in a preferred antiparallel conformation (tilt ≥ 130°). Mutated dimers in contrast exhibit a significant increased flexibility. The angles were determined over the last 50 ns of the trajectories.

**Figure S6: Mutant dimers adopt an increased flexibility.**

(**a**) Number of orientation transitions between the intervals as indicated by vertical blue lines in the inset. The transitions were determined over the last 50 ns of the coarse-grained simulations. *Inset*: histogram of the tilt angles, determined over the last 50 ns of the simulations. (**b**) Boxplots of the number of transitions between relative orientations as shown in (a) indicate an increased flexibility of mutated CTH-3LA dimers as compared to wild type dimers. The thick line in each box represents the median, which is 0 for the wild type and 4.5 for the mutants. Within the red and green box lie 50% of the data points and the horizontal line at the end of the vertical dashed line represents the last data point, which is not larger than 1.5 times the box length from the box. Circles represent data points that exceed this threshold.

**Figure S7: Synthetic CTH peptides inhibit TPC1 *in vivo*.**

Overlay of current traces recorded from excised patches of wild type Col-0 vacuoles during voltage steps to +87 mV, starting from a holding potential of -53 mV. Traces were recorded in 5 s intervals in the presence of the buffer control (a), or during application of the synthetic CTH peptide at a final concentration of 15 µM (b). The presence of the CTH peptide inhibited the current within 10 s (red traces are shown at about 5 and 10 s after application). A similar current inhibition was observed in 6 independent experiments.

**Supplementary Tables**

**Table S1: Details of plasmid constructions.**

| **Construct** | **Primers used** | **Comments** |
| --- | --- | --- |
| **For C-terminal eGFP-fusion with p2GWF7,0 [93]** | | |
| AtTPC1-ΔC_1-678_ | 1; 2 |  |
| AtTPC1-ΔC29_1-704_ | 3; 4 |  |
| AtTPC1-ΔC8_1-725_ | 3; 5 |  |
| AtTPC1-3LA | 3; 6; 7; 8 | L714,715,719A |
| AtTPC1-3LP | 3; 8; 9; 10 | L714,715,719P |
| AtTPC1_1-733_ | 1; 11 | [11,94] |
| AtTPC1-S706A | 3; 8; 12; 13 |  |
| AtTPC1-S706D | 3; 8; 14; 15 |  |
| **For Bimolecular fluorescence complementation** | | |
| AtTPC1_1-733_ | see above | For pDEST–^GW^VYCE and pDEST–^GW^VYNE |
| AtTPC1+st_1-733_ | 3; 16 | For pDEST–VYCE(R)^GW^ and pDEST–VYNE(R)^GW^ |
| AtTPC1-3LP | see above | For pDEST–^GW^VYCE and pDEST–^GW^VYNE |
| **For Co-IP; N-terminal fusion of 6xMyc- or Flag-tag** | | |
| AtTPC1-Ct+eGFP+st | 17; 18 | Template: AtTPC1/p2GWF7,0 |
| AtTPC1-3LP-Ct+eGFP+st | 17; 18 | Template: AtTPC1-3LP/p2GWF7,0 |

Table S2: The primers used for PCR.

| **Number** | **Sequence 5’ 3’** |
| --- | --- |
| **1** | CACCTCTAGATGGAAGACCCG |
| **2** | AGTAAAGAACGCCTCCAAGACAAAG |
| **3** | CACCATGGAAGACCCGTTGATTGG |
| **4** | GAGCTCCGACCCTGCAGATCGACG |
| **5** | GAGCTCTTTGCTGAGTTCATCACC |
| **6** | CAGAGAGTTGATACAGCTGCTCATCATATGGCGGGTGATGAACTCAGC |
| **7** | GCTGAGTTCATCACCCGCCATATGATGAGCAGCTGTATCAACTCTCTG |
| **8** | TCTAGATGTGTCAGAAGTGGAACAC |
| **9** | GTTCATCACCCGGCATATGATGAGGAGGTGTATCAACTC |
| **10** | GAGTTGATACACCTCCTCATCATATGCCGGGTGATGAAC |
| **11** | GAGCTCTGTGTCAGAAGTGG |
| **12** | GCGTCGATCTGCTGGGTCGAAGGCTCGGAGTCAG |
| **13** | CTGACTCCGAGCCTTCGACCCAGCAGATCGACGC |
| **14** | GCGTCGATCTGCTGGGTCGAAGGATCGGAGTCAG |
| **15** | CTGACTCCGATCCTTCGACCCAGCAGATCGACGC |
| **16** | CTCGAGTTATGTGTCAGAAGTGG |
| **17** | CACCGAATTCAATGGAGGCGTTCTTTACTGAGCTGGATC |
| **18** | CTCGAGTTACTTGTACAGCTCGTCCATGC |
